# Incidence of synchronous appendiceal neoplasm in patients with colorectal cancer and its clinical significance

**DOI:** 10.1186/1477-7819-7-51

**Published:** 2009-06-02

**Authors:** Varut Lohsiriwat, Akkrarash Vongjirad, Darin Lohsiriwat

**Affiliations:** 1 Department of Surgery, Faculty of Medicine Siriraj Hospital, Mahidol University, Bangkok 10700, Thailand

## Abstract

**Background:**

The aims of this study were to evaluate the incidence of synchronous appendiceal neoplasm in patients with colorectal cancer, and to determine its clinical significance.

**Methods:**

Pathological reports and medical records were reviewed of patients with colorectal adenocarcinoma who underwent oncological resection of the tumor together with appendectomy at the Faculty of Medicine Siriraj Hospital, Mahidol University, Thailand between September 2000 and April 2008.

**Results:**

This study included 293 patients with an average age of 62 years (range 19–95) and 51 percent were male. Of the patients studied, 228 (78 percent) had right hemicolectomy, whereas the others (22 percent) had surgery for left-sided colon cancer or rectal cancer. One patient (0.3 percent) had epithelial appendiceal neoplasm (mucinous cystadenoma) and 3 patients (1.0 percent) had metastatic colorectal cancer in the mesoappendix. However, the presence of synchronous appendiceal tumors and/or metastasis did not alter postoperative management, as these patients had received adjuvant therapy and were scheduled for surveillance program because of nodal involvement.

**Conclusion:**

The incidence of synchronous primary appendiceal neoplasm and secondary (metastatic) appendiceal neoplasm in colorectal cancer patients was 0.3 and 1.0 percent, respectively. However, these findings did not change the postoperative clinical management.

## Background

Synchronous colorectal cancer (CRC) has been reported in 0.6–1.4 percent of patients and metachronous CRC in 1–8 percent of patients [1]. Any neoplastic change of the colon and rectum could possibly affect the appendix because the appendix is derived embryologically from the large intestine and has a similar mucosal pattern to the colon and rectum. The histological features of appendiceal adenocarcinoma are also identical to those of colorectal adenocarcinoma [2]. Moreover, it has been reported that almost a quarter of patients with appendiceal cancer are found to have synchronous or metachronous neoplasms elsewhere in the large intestine [2,3].

Although there have been increasing advances in both endoscopy and radiology, the appendiceal mucosa remains inaccessible and the accuracy of preoperative diagnosis of appendiceal neoplasm is still poor. During an operation, a correct diagnosis is made in less than half of the cases [4]. Several case reports of synchronous appendiceal tumors in CRC patients have been published in the literature [5-9], but only one study has explored its incidence which is about 4 percent of CRC patients having synchronous appendiceal neoplasm [7]. Given the difficulty in diagnosis of appendiceal tumors and the certain risk of synchronous and metachronous neoplasm of the appendix, the question of whether an incidental appendectomy should be performed in CRC patients has been raised [5,7].

In attempt to address this question, it is first essential to know the incidence and the biological significance of synchronous appendiceal tumors in CRC patients. The aims of this study were to evaluate the incidence of synchronous appendiceal neoplasm in patients with resectable CRC in a university hospital, and to determine the clinical significance of these findings.

## Methods

Pathological reports and medical records were retrospectively reviewed of patients with colorectal adenocarcinoma who underwent oncological resection of the tumor together with appendectomy at the Department of Surgery, Faculty of Medicine Siriraj Hospital, Mahidol University, Bangkok, Thailand between September 2000 and April 2008. Patients were excluded if they had familial adenomatous polyposis syndrome or had had a previous appendectomy, or if there had been direct invasion of CRC to the appendix. Patients receiving neoadjuvant therapy were also excluded. Notably, there were about 100–120 operations for CRC per annum in our unit, and one-third of them were for right-sided colon cancer.

The appendix is normally a part of the specimen removed in patients with right hemicolectomy. All the appendices removed along with the right colon specimen were systematically analyzed. Incidental appendectomy had also been performed in patients who had undergone left hemicolectomy or rectal resection at the discretion of the surgeon. We tended to perform incidental appendectomy if the patient was younger than 45 years, or there was a fecalith in the appendix. Histopathological study of the appendix included gross and microscopic examination. Specimens were sectioned at the tip, body, and base of the appendix as well as other suspicious lesions. All specimens were examined by a consultant or senior pathologist.

The site of the primary tumor, type of operation, and pathological staging of CRC were noted. Macroscopic and microscopic features of the appendix were also recorded. The presence of synchronous appendiceal tumors and/or metastasis was correlated with follow-up data to demonstrate the clinical significance of these findings. The study was approved by the Institutional Ethics Committee.

## Results

Two hundred and ninety-three patients were included in this study. The patients studied had an average age of 62 years (range 19–95) and 51 percent were male. Of the patients studied, 228 (78 percent) had a right hemicolectomy, 45 (15 percent) had a left hemicolectomy, and 20 (7 percent) had surgery for rectal cancer. One patient (0.3 percent) had epithelial appendiceal neoplasm (mucinous cystadenoma), and 3 patients (1.0 percent) had metastatic colorectal cancer in the mesoappendix (Table 1). All metastatic lesions were mucinous adenocarcinoma. Clusters of metastatic cancerous cells were 1–5 mm in diameter; primarily located in the subserosal area. All appendices with neoplasms did not appear abnormal in the preoperative imaging and during the intraoperative examination. However, the case of metastases in the mesoappendix from a descending colon cancer was associated to other peritoneal implants.

**Table 1:** Patients' characteristics, details of primary colorectal cancer, and pathological results of synchronous appendiceal neoplasm

Age (years)/Gender	Primary tumor	Type of surgery	Staging of primary tumor	Appendix abnormality
59 M	cecum	RH	T3N2	Metastatic adenocarcinoma in the mesoappendix
62 F	ascending colon	RH	T3N2	metastatic adenocarcinoma in the mesoappendix
48 M	descending colon	LH	T3N1	Metastatic adenocarcinoma in the mesoappendix
64 F	rectum	APR	T3N2	mucinous cystadenoma

Abbreviation: RH (right hemicolectomy), LH (left hemicolectomy), APR (abdominoperineal resection)

There was no specific morbidity that could be attributed to incidental appendectomy in the present study. The presence of synchronous appendiceal tumors and/or metastasis did not alter postoperative management, as these patients had received adjuvant therapy and were scheduled for surveillance program because of nodal involvement.

## Discussion

The question of whether an incidental appendectomy should be performed in CRC patients has been raised due to the difficulty in diagnosis of appendiceal tumors and the certain risk of synchronous and metachronous neoplasm of the appendix [5,7]. Little is known about the incidence of appendiceal neoplasm in CRC patients. To the best of our knowledge, there was only one study determining such an incidence [7]. Khan and Moran retrospectively reviewed 169 CRC patients who underwent CRC surgery and removal of the appendix in Basingstoke, United Kingdom. They reported 4.1 percent synchronous primary appendiceal neoplasm in these patients, and mucinous cystadenoma was the most common neoplasm found [7]. Furthermore, these authors suggested performing incidental appendectomy in all CRC patients. Meanwhile, Albright et al determined the cost-effectiveness of interval appendectomy in patients who undergo curative resection for CRC and found that the benefit in cost was only realized for patients younger than 45 years of age [5].

Synchronous CRC and appendiceal tumors have been observed in high-risk patients such as those with long-standing ulcerative colitis [10]. Moreover, patients with rectal cancer had a slightly higher rate of synchronous appendiceal tumors than those with right-sided colon cancer [7]. However, absent from the literature are such studies in Asian population, in which the incidence of synchronous appendiceal neoplasms in CRC patients could be different.

The present study in Thailand demonstrates that the incidence of synchronous primary appendiceal neoplasm and secondary (metastatic) appendiceal neoplasm in patients with resectable CRC is 0.3 and 1.0 percent, respectively. One possible explanation for the low incidence of synchronous primary neoplasm of the appendix in CRC patients in the present study could be that tumorigenesis of the appendix and other parts of the large intestine are not the same. Appendiceal mucosa is not directly exposed to potential carcinogens in fecal material as the colorectal mucosa is. Epidemiological study revealed that appendiceal tumors account for 0.4–1 percent of alimentary tract cancers and are found in 0.7–1.7 percent of appendectomy specimens [11], whilst CRC is the most common gastrointestinal malignancy [12]. Also, the peak incidence of appendiceal neoplasm is in patients in their early forties, 20-year younger than that of CRC [13]. It is possible that the incidence of appendiceal tumors in Asian population is different from that of Western population [14], and thus the incidence of synchronous appendieal neoplasms in CRC patients could vary among various ethnic and geographic backgrounds. Lastly, in view of the examination of the specimens, different protocols of tissue section and methods of histopathological examination may lead to differences in the incidence percentages.

The unexpected finding in the present study was that one percent of CRC patients had metastatic lesions in the mesoappendix. This finding was fairly consistent with a previous study by Albright and his colleagues [5]. They reported 2 cases of metastatic implants to the appendix from routine interval appendectomy in 210 patients with intraabdominal malignancy; accounting for 0.95%. One case was secondary to a sigmoid cancer with limited peritoneal carcinomatosis while the other was secondary to an ovarian adenocarcinoma. As the mesoappendix encloses the appendiceal artery and vein, together with lymphatic vessels and lymph node, metastasis to the mesoappendix could occur via the lymphatic, hematogenous or transcoelomic route. With regard to the transcoelomic route, CRC may spread throughout the peritoneum either via the subperitoneal lymphatic drainage or by viable cells being shed from the serosal surface of a tumor [15]. This is supported by the observations that 14.6 percent of CRC had positive cytology for cancer cells on the peritoneal or perirectal surface of the bowel, particularly in those with extensive lymphatic involvement, poorly differentiated tumors, or liver metastases [16]. Metastasis to the appendix has been reported in both gastrointestinal and non-gastrointestinal malignancies such as gastric [17], pancreatic [18], ovarian [5,19], cervical [20], nasopharyngeal [21], breast [22], and lung [23].

A limitation of this single-center study is a relatively small sample size, particularly those with left-sided colon cancer and rectal cancer. Considering this reason, a larger number of incidental appendectomy in patients with left-sided colon cancer and rectal cancer are warranted to verify our findings. Besides, this review has some limitations which are mainly inherent to a retrospective study and to different clinical judgments of surgeons. There could be a selection bias to perform incidental appendectomy as we did not have specific criteria for performing appendectomy in CRC patient at our institute. It is possible that the appendix was more likely to be removed because of its abnormal appearance. In order to determine the true incidence of synchronous appendiceal neoplasms, a cohort or prospective study of patients where the appendix is always removed (either by necessity in a right hemicolectomy specimen; or as a protocol where the appendix is always removed in CRC patients) is required.

## Conclusion

Based on this study, the incidence of synchronous primary appendiceal neoplasm and secondary (metastatic) appendiceal neoplasm in CRC patients was 0.3 and 1.0 percent, respectively. These findings did not change the postoperative clinical management.

## Abbreviations

APR: abdominoperineal resection; CRC: colorectal cancer; LH: left hemicolectomy; RH: right hemicolectomy.

## Competing interests

The authors declare that they have no competing interests.

## Authors' contributions

VL was the principal investigator who participated in research design, analyzed the data, and prepared the manuscript. AV contributed to acquisition and analysis of data. DL conceived of the study, participated in its design and coordination, and helped to draft the manuscript. All authors read and approved the final manuscript.
